# Hemodynamic Response to Laryngoscopy and Endotracheal Intubation With Conventional Macintosh Laryngoscope Versus C‐MAC Video Laryngoscope in Patients Undergoing Elective Coronary Artery Bypass Grafting: A Randomized Controlled Trial

**DOI:** 10.1155/anrp/2771364

**Published:** 2026-06-17

**Authors:** Sehrish Khan, Malika Hameed, Waleed Razi Khan, Muhammad Saad Yousuf, Khalid Samad

**Affiliations:** ^1^ Department of Anesthesiology, Aga Khan University, Karachi, Pakistan, aku.edu

**Keywords:** airway management, coronary artery bypass, hemodynamics, intubation, laryngoscopes, laryngoscopy

## Abstract

**Background:**

Laryngoscopy and endotracheal intubation evoke significant hemodynamic responses, particularly in patients undergoing coronary artery bypass grafting (CABG) who have compromised cardiac reserves. This study aimed to compare the hemodynamic effects of the C‐MAC video laryngoscope (VL) with those of the conventional Macintosh laryngoscope (MC) in patients undergoing elective CABG.

**Methods:**

This randomized controlled trial included 86 patients scheduled for elective CABG, who were randomly assigned to intubation with either the MC group or the C‐MAC VL group. Hemodynamic parameters, including heart rate, systolic, diastolic, and mean arterial pressures, were recorded at five time points (prelaryngoscopy baseline to 10 min postintubation). Secondary outcomes included the intubation success rate, duration of laryngoscopy and intubation, glottic visualization, and complications.

**Results:**

Two‐way repeated measures ANOVA revealed no significant group effect or group‐by‐time interaction for any hemodynamic variable (all *p* > 0.05), confirming equivalent hemodynamic profiles across all time points. A significant time effect was observed for all variables (all *p* < 0.001). First‐attempt intubation success was 88.4% (VL) versus 74.4% (MC) (*p* = 0.166). The C‐MAC provided significantly better glottic visualization (modified CL grade, *p* < 0.001). No major complications occurred in either group.

**Conclusion:**

In patients undergoing CABG, C‐MAC VL and conventional MC result in similar hemodynamic responses. The choice of laryngoscope may be guided by airway considerations and operator experience rather than anticipated cardiovascular effects.

**Trial Registration:** Clinicaltrials.gov_identifier: NCT04433884

## 1. Introduction

Cardiac surgery, such as coronary artery bypass grafting (CABG), typically requires general anesthesia, which involves laryngoscopy followed by endotracheal intubation. The force exerted during laryngoscopy is the primary factor responsible for the mechanical stimulation of the supraglottic region and stretch receptors in the respiratory tract. These procedures can evoke significant stress responses, including tachycardia, systemic hypertension, arrhythmias, and increased intracranial pressure [1, 2]. Patients undergoing CABG often have limited cardiac reserves, making them particularly vulnerable to the hemodynamic fluctuations induced by these procedures. These fluctuations can lead to adverse effects, potentially precipitating a myocardial supply–demand imbalance [3].

Additionally, CABG patients frequently exhibit airway abnormalities due to diabetes mellitus and age‐related arthritic changes. These can include reduced neck mobility, dental issues, and limited mouth opening, which increase the incidence of difficult laryngoscopy in this patient population compared with that in general surgical patients [4].

Minimizing the stress response during airway manipulation is crucial for reducing hemodynamic disturbances and the risk of perioperative myocardial ischemia in cardiac patients with compromised cardiorespiratory reserves. While various pharmacological agents have been used to attenuate this response, there is limited information on the impact of different laryngoscope blade designs [5].

In recent years, video laryngoscopes (VLs), particularly the C‐MAC VL introduced by Karl Storz in 1990, have gained popularity. This device features a modified Macintosh blade and provides both a direct laryngoscopic view and a visual display on a screen, facilitating airway management in both normal and difficult scenarios [6].

Previous studies, such as those by Sarkilar et al., have suggested that the C‐MAC could be an effective alternative airway tool for major cardiac surgery patients with poor glottic views via direct laryngoscopy. While the C‐MAC may still require the use of a stylet and external cricoid pressure to optimize the view, it offers a viable alternative for managing difficult airways in this patient group [7].

Despite the potential benefits, data comparing the circulatory responses to the C‐MAC VL with the conventional Macintosh laryngoscope (MC) in patients undergoing CABG are limited [7, 8].

Given the potential impact of airway devices on hemodynamic responses, this randomized controlled trial was designed to evaluate the hemodynamic response to laryngoscopy and tracheal intubation in patients undergoing elective CABG using either a conventional MC or the C‐MAC VL.

## 2. Methods

### 2.1. Study Design and Participants

The study was conducted in the cardiac operating rooms of The Aga Khan University Hospital after obtaining approval from The Aga Khan University ethics review committee (2020‐3278‐9046). The study was subsequently registered at https://www.clinicaltrials.gov and conducted in accordance with the principles of the Declaration of Helsinki.

This randomized controlled trial included patients aged 35–65 years who were scheduled for elective CABG. Patients were excluded if they had a body mass index (BMI) greater than 35 kg/m2, significant left main coronary artery critical disease, left heart failure, a left ventricular ejection fraction of less than 35%, or a history of recent myocardial infarction (MI) or unstable angina. Patients with two or more predictors of difficult intubation (Mallampati Class III or IV, upper lip bite test class C, thyromental distance less than 6 cm, mouth opening less than three finger breadths, and limited neck mobility) were also excluded from the study [9].

### 2.2. Randomization

After obtaining written informed consent, patients were randomly allocated to either the MC group or the VL (C‐MAC) group using a sealed opaque envelope technique. Each envelope contained the name of either the C‐MAC or MC. It was opened prior to the induction of anesthesia, and the designated laryngoscope was then assigned to the anesthesia team. The envelopes were prepared using a computer‐generated randomization table.

### 2.3. Anesthesia Protocol

All patients were premedicated with oral midazolam (7.5 mg) 45–60 min before anesthesia. Upon entering the operating room, standard monitoring was applied, which included electrocardiography (ECG) and pulse oximetry (SpO2). A radial arterial line was inserted under local anesthesia and a wide‐bore IV cannula was established before anesthesia induction. The anesthesia technique was standardized across both groups.

Following preoxygenation, anesthesia induction was done with midazolam (0.02–0.05 mg·kg^−1^), propofol (0.5–1 mg·kg^−1^), and fentanyl (5 μg·kg^−1^), followed by cis‐atracurium (0.15 mg·kg^−1^) for muscle relaxation. Manual ventilation was performed using isoflurane (1% end‐tidal) in oxygen via a facemask for 3 min.

### 2.4. Intubation Procedure

Intubation was achieved by inserting an endotracheal tube using either a MC or a C‐MAC VL. No rapid sequence induction (RSI) was performed, and neuromuscular monitoring was not used. No patient received opioid premedication prior to arrival in the operating room, and none were chronic opioid users. All intubations were performed by a consultant anesthesiologist or a senior resident with at least three years of experience who had performed a minimum of 20 intubations with a VL. Postinduction, central venous catheters and, if indicated, Swan–Ganz catheters were placed for hemodynamic monitoring. All patients underwent mechanical ventilation for the duration of the surgery, with anesthesia maintained using isoflurane (end‐tidal concentration 1%–1.5%) in oxygen. During the 10‐min data collection period postintubation, no additional medications were given, nor were any other procedures performed. Subsequent patient management was left to the discretion of the attending anesthesiologist.

### 2.5. Primary Outcome

The primary outcome of the study was the hemodynamic response to laryngoscopy and endotracheal intubation, which was assessed by measuring heart rate, systolic arterial pressure, diastolic arterial pressure, and mean arterial pressure (MAP) at five predefined time points. These included T1 = baseline‐immediately before laryngoscopy, T2 = after endotracheal intubation, T3 = 1 min after endotracheal intubation, T4 = 5 min after endotracheal intubation, and T5 = 10 min after endotracheal intubation. These parameters were continuously monitored via the radial arterial line using the anesthesia monitoring system, with values at each time point representing instantaneous readings. No additional averaging interval was applied.

### 2.6. Secondary Outcomes

Secondary outcomes, which included the duration of laryngoscopy (DOL) and intubation (DOLI), glottic view during laryngoscopy, the number of intubation attempts, the requirement for optimal laryngeal external manipulation (OLEM) during intubation, and complications (changes in rhythm, oral bleeding, lacerations, and dental injury during intubation), were also recorded.

DOL was defined as the time from oral placement of the laryngoscope blade to obtaining the best glottic view. For the evaluation of glottic view during laryngoscopy, the modified Cormack and Lehane Scoring System (m‐CL) [10] and the percentage of the glottis opening (POGO) score were used [11].

Duration of intubation (DOI) was defined as the time interval between oral placement of the endotracheal tube and the attainment of a tracing of end tidal CO_2_ waveform after intubation and initiation of mechanical ventilation.

Failure to intubate was defined as the inability to intubate after three attempts. An alternative technique was used in cases of failure at the discretion of the anesthetist. These patients were excluded from the study. The DOLI was taken as the sum of all intubation attempts.

### 2.7. Sample Size

The sample size calculation was based on the previous study by Kanchi M. et al., which reported a MAP of 84 ± 16 mmHg in patients intubated with a Macintosh blade and 70.6 ± 16 mmHg in those intubated using a VL [8]. A sample size of 43 patients in each group was required to achieve 80% power to detect an absolute difference of 10–20 mmHg in MAP with a 5% type I error.

### 2.8. Statistical Analysis

Statistical analyses were performed using RStudio 4.1.2 (R Foundation for Statistical Computing). Continuous variables are expressed as mean±standard deviation (SD) and categorical variables as frequency and percentage. The normality of continuous variables was assessed using the Shapiro–Wilk test. Baseline quantitative variables were compared between groups using an independent samples *t*‐test, while categorical variables were compared using the chi‐squared test or Fisher’s exact test as appropriate. Hemodynamic variables (heart rate, systolic blood pressure, diastolic blood pressure, and MAP) measured at multiple time points (T1–T5) were analyzed using two‐way repeated measures analysis of variance (ANOVA), with group (Macintosh vs. C‐MAC) as the between‐subjects factor and time (T1–T5) as the within‐subjects factor. The Group x Time interaction was also examined. A *p* value < 0.05 was considered statistically significant.

## 3. Results

**FIGURE 1:**
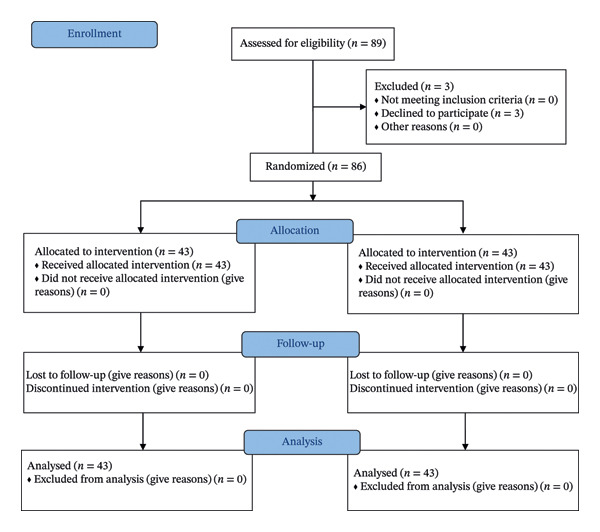
CONSORT flow diagram.

A total of 89 participants were assessed for eligibility, of which 3 were excluded as they declined to participate in this trial. Following this, a total of 86 patients undergoing elective CABG were included in this study after providing written informed consent (Figure 1). Of these, 43 patients each were randomly allocated to the MC and VL groups.

The demographic data are presented in Table 1 and were found to be comparable, with no significant differences observed between the two groups in terms of age, gender, BMI, incidence of hypertension, incidence of diabetes mellitus, LV ejection fraction, and Mallampati score.

**TABLE 1:** Patients’ demographic and other details.

Variable	Total (*N* = 86)	MC group (*N* = 43)	VL group (*N* = 43)	*p*‐value
Age (years)				
Mean ± SD	57.8 ± 9.59	59.3 ± 10.3	56.4 ± 8.73	0.168
Sex				
Male	71 (82.6%)	33 (76.7%)	38 (88.4%)	0.256
Female	15 (17.4%)	10 (23.3%)	5 (11.6%)	
Body Mass Index (BMI) (kg/m^2^)				
Mean ± SD	28.7 ± 4.72	28.8 ± 4.21	28.7 ± 5.25	0.961
American Society of Anesthesiologists (ASA) status				
IV	86 (100%)	43 (100%)	43 (100%)	1.00
Ejection fraction (%)				
Mean ± SD	47.3 ± 10.1	48.1 ± 9.09	46.5 ± 11.1	0.474
Comorbidity (%)				
Hypertension	70 (81.4%)	36 (83.7%)	34 (79.1%)	0.782
Diabetes mellitus	45 (52.3%)	24 (55.8%)	21 (48.8%)	0.666
Duration of laryngoscopy (seconds)				
Mean ± SD	60.4 ± 29.9	65.8 ± 32.1	54.9 ± 26.7	0.092
Duration of intubation (seconds)				
Mean ± SD	32.7 ± 23.1	37.5 ± 25.8	28.0 ± 19.1	0.058
Duration of laryngoscopy and intubation (seconds)				
Mean ± SD	93.1 ± 49.8	103 ± 53.1	83.0 ± 44.5	0.058

Abbreviations: MC, Macintosh laryngoscope; SD, standard deviation; VL, video laryngoscope.

Airway management related procedural variables are also summarized in Table 1. The duration of laryngoscopy, intubation, and total procedural time (laryngoscopy and intubation) showed a tendency toward longer durations in the MC group compared with the VL group. However, these differences did not reach statistical significance (*p* = 0.058–0.092).

Hemodynamic parameters recorded at five time points (T1–T5) are presented in Table 2. Two‐way repeated measures ANOVA revealed no statistically significant “Group effect” for any hemodynamic variable, indicating that the overall hemodynamic levels did not differ between the MC and VL groups across the measurement period (heart rate: F (1.84) = 0.157 and *p* = 0.693; systolic blood pressure: F (1.84) = 1.064 and *p* = 0.305; diastolic blood pressure: F (1.84) = 2.543 and *p* = 0.115; MAP: F (1.84) = 1.198 and *p* = 0.277). A significant “Time effect” was observed for all four variables (all F (4.336) > 13 and *p* < 0.001), reflecting the expected physiological changes in hemodynamics during and after laryngoscopy and intubation in both groups. Critically, the “Group x Time interaction” was not statistically significant for any variable (heart rate: F (4.336) = 0.451 and *p* = 0.772; systolic blood pressure: F (4.336) = 1.154 and *p* = 0.331; diastolic blood pressure: F (4.336) = 1.837 and *p* = 0.121; MAP: F (4.336) = 1.332 and *p* = 0.258), confirming that the hemodynamic trajectories of the two groups were parallel and equivalent across all time points. No post hoc comparisons were performed as no significant Group x Time interaction was detected.

**TABLE 2:** Hemodynamic (mean ± SD)‐related changes parameters.

**Time**	**Heart rate (bpm)**		**Systolic BP (mmHg)**		**Diastolic BP (mmHg)**		**MAP (mmHg)**	
	**MC (*n* = 43)**	**VL (*n* = 43)**	**MC (*n* = 43)**	**VL (*n* = 43)**	**MC (*n* = 43)**	**VL (*n* = 43)**	**MC (*n* = 43)**	**VL (*n* = 43)**

T1	74.9 ± 15.0	75.1 ± 13.6	143.8 ± 28.2	145.7 ± 21.8	76.3 ± 14.9	81.4 ± 10.9	99.0 ± 19.9	102.1 ± 12.5
T2	72.1 ± 14.3	69.5 ± 12.6	120.1 ± 30.9	130.8 ± 25.6	64.2 ± 19.0	72.5 ± 15.5	83.3 ± 22.7	91.3 ± 16.8
T3	77.7 ± 15.1	77.3 ± 13.8	140.7 ± 27.0	143.6 ± 27.6	76.2 ± 18.3	77.6 ± 14.6	96.9 ± 21.9	98.6 ± 17.8
T4	73.5 ± 15.1	72.7 ± 11.5	122.0 ± 30.1	128.3 ± 21.0	65.7 ± 17.5	70.0 ± 12.5	85.4 ± 20.1	89.0 ± 13.5
T5	71.2 ± 13.5	69.7 ± 11.3	118.3 ± 22.4	119.9 ± 19.9	64.7 ± 11.9	66.8 ± 11.6	83.3 ± 13.7	83.3 ± 11.9
ANOVA	Group: F (1.84) = 0.157, *p* = 0.693 Time: F (4.336) = 13.594, *p* < 0.001 Interaction: F (4.336) = 0.451, *p* = 0.772		Group: F (1.84) = 1.064, *p* = 0.305 Time: F (4.336) = 40.706, *p* < 0.001 Interaction: F (4.336) = 1.154, *p* = 0.331		Group: F (1,84) = 2.543, *p* = 0.115 Time: F (4,336) = 34.000, *p* < 0.001 Interaction: F (4,336) = 1.837, *p* = 0.121		Group: F (1,84) = 1.198, *p* = 0.277 Time: F (4,336) = 33.510, *p* < 0.001 Interaction: F (4,336) = 1.332, *p* = 0.258	

*Note:* Values are mean±standard deviation. MC = Macintosh laryngoscope group; VL = C‐MAC video laryngoscope group; mmHg = millimeters of mercury. T1 = baseline (immediately before laryngoscopy); T2 = immediately after intubation; T3 = 1 min postintubation; T4 = 5 min postintubation; T5 = 10 min postintubation. Hemodynamic parameters were analyzed using two‐way repeated measures ANOVA with Group (MC vs. VL) as the between‐subjects factor and Time (T1‐T5) as the within‐subjects factor; the Group x Time interaction was also assessed. No statistically significant Group effect or Group x Time interaction was detected for any variable (all *p* > 0.05). The significant Time effect reflects the expected physiological hemodynamic response to intubation in both groups combined. Sphericity was assessed using Mauchly’s test; Greenhouse–Geisser correction was applied where violated.

Abbreviations: BP, blood pressure; bpm = beats per minute; MAP, mean arterial pressure.

First‐attempt intubation success was 88.4% in the VL group versus 74.4% in the MC group (*p* = 0.166) (Table 3). There were no changes in rhythm, oral bleeding, lacerations, or dental injury during intubation reported during the study period in either of the groups.

**TABLE 3:** Airway and intubation related parameters.

Variable	MC group (*N* = 43)	VL group (*N* = 43)	*p* value
Mallampati Class			0.926
I	10 (23.3%)	8 (18.6%)	
II	24 (55.8%)	27 (62.8%)	
III	8 (18.6%)	7 (16.3%)	
IV	1 (2.33%)	1 (2.33%)	
Upper lip bite test			0.975
A	27 (62.8%)	26 (60.5%)	
B	15 (34.9%)	16 (37.2%)	
C	1 (2.33%)	1 (2.33%)	
Thyromental distance			1.000
Less than 6 cm	3 (6.98%)	2 (4.65%)	
More than 6 cm	40 (93.0%)	41 (95.3%)	
Modified Cormac Lehane Grade			< 0.001
I	18 (41.9%)	22 (51.2%)	0.401
II	5 (11.6%)	19 (44.2%)	0.001
IIa	12 (27.9%)	2 (4.65%)	0.003
IIb	5 (11.6%)	0 (0%)	0.021
III	3 (6.98%)	0 (0%)	0.078
Percentage of glottic opening (POGO) score			0.310
Grade I	2 (4.65%)	0 (0%)	0.153
Grade II	23 (53.5%)	21 (48.8%)	0.660
Grade III	18 (41.9%)	22 (51.2%)	0.389
No. of attempts of intubation			
1	32 (74.4%)	38 (88.4%)	0.166
> 1	11 (25.6%)	5 (11.6%)	
Optimal laryngeal external manipulation (OLEM)			
Yes	17 (39.5%)	15 (34.9%)	0.823
No	26 (60.5%)	28 (65.1%)	

Abbreviations: MC, Macintosh laryngoscope; VL, video laryngoscope.

## 4. Discussion

Our study found no significant differences in the hemodynamic responses between patients intubated for CABG using the conventional MC and those intubated with the C‐MAC VL. Two‐way repeated measures ANOVA demonstrated no significant group effect or group–time interaction for any hemodynamic variable, indicating that both techniques produced comparable overall hemodynamic profiles and similar temporal trends following intubation. However, a significant time effect was observed for all variables, reflecting the expected physiological response to laryngoscopy and endotracheal intubation. Laryngoscopy and tracheal intubation are known to evoke significant sympathetic responses, including increases in blood pressure, heart rate, and catecholamine release, especially in patients with cardiac conditions. However, the magnitude of these hemodynamic perturbations can vary depending on the type of laryngoscope used. Direct laryngoscopy, as performed with the Macintosh blade, typically involves greater tissue manipulation, which may exacerbate sympathetic stimulation [12].

The results of our study align with a previous study by Sarkilar et al., which also reported similar hemodynamic outcomes with the use of a VL and MC in cardiac surgery patients [7]. Their study was conducted on a diverse group of cardiac surgery patients with varying cardiac conditions, including those undergoing CABG and valve surgeries.

Aggarwal et al. compared the hemodynamic response to orotracheal intubation using the C‐MAC, Macintosh, and McCoy laryngoscopes in American Society of Anesthesiologists (ASA) physical status Classes I and II patients undergoing elective surgeries [13]. Their intergroup analysis revealed that the hemodynamic response to intubation between the C‐MAC and MC groups was not statistically significant. Although their study population did not include cardiac patients, their findings are consistent with the results of our study.

Similarly, the study by Kanchi et al. also reported that despite the longer intubation time with VL, there was no significant difference in the hemodynamic responses between conventional laryngoscopy and VL in patients undergoing CABG [8].

In contrast, Buhari et al. reported that C‐MAC laryngoscopy elicited a higher hemodynamic response, particularly with increased SBP and HR at 1‐ and 3‐min postintubation, compared with the MC [14]. The discrepancy between our findings and Buhari et al.’s results may be attributed to differences in patient populations. While Buhari et al. studied ASA I patients undergoing elective surgeries, our study focused on cardiac surgery patients who were already on preoperative beta blockers and other cardiovascular medications, potentially attenuating the hemodynamic response.

The absence of significant hemodynamic differences between the groups in our study could be attributed to several factors. First, the standardized anesthesia protocol used across both groups, including the administration of fentanyl and midazolam, likely minimized the cardiovascular response to intubation. This is consistent with findings by Sarkilar et al., who noted that pharmacological agents such as fentanyl can effectively blunt the stress response during intubation [7]. Additionally, the experience level of the anesthesiologists performing the procedures may have further reduced the variability in intubation techniques and their associated hemodynamic consequences.

The DOLI is directly associated with the magnitude of the cardiovascular response [14]. Previous studies by Sarkilar et al. and Kanchi et al. both observed longer intubation times with VLs in cardiac patients [7, 8]. In contrast, our study showed a tendency toward shorter laryngoscopy and intubation times with the C‐MAC VL, though none of these differences reached statistical significance (*p* = 0.058–0.092). These findings may suggest a trend toward improved procedural efficiency with video laryngoscopy but should be interpreted with caution. One plausible explanation for this finding is the increased familiarity with video laryngoscopy among our residents and faculty during the COVID‐19 pandemic in 2020. During this period, VLs were preferred for all patients, regardless of their COVID‐19 status, as part of infection control measures. The experience gained from this widespread use likely contributed to a reduction in laryngoscopy and intubation times, as VLs are known to have a learning curve [15, 16]. This observation is consistent with a broader global trend, as a large multicenter survey reported a substantial increase in VL use during the COVID‐19 pandemic, rising from 24.1% to 43.1% of the cases across participating institutions [17].

Our findings have important clinical implications, particularly for anesthesiologists managing patients with limited cardiac reserves. The choice between a conventional MC and the C‐MAC VL may be based more on airway management considerations and an anesthesiologist’s familiarity rather than on expected reductions in hemodynamic disturbances. For patients with difficult airways, the C‐MAC offers superior visualization, which may reduce the need for multiple intubation attempts and associated complications. However, for patients with stable hemodynamics and predictable airways, either device can be safely used without significant differences in cardiovascular outcomes.

Despite the strengths of our study, including its randomized design and focus on a high‐risk patient population, it has limitations. The sample size was relatively small, and we excluded patients with more severe left ventricular dysfunction, which may have influenced the hemodynamic outcomes. Further studies involving larger patient populations and broader cardiac surgical cohorts are warranted to better define the potential clinical advantages of video laryngoscopy in high‐risk cardiac patients.

## 5. Conclusion

In conclusion, our findings suggest that in patients undergoing elective CABG, endotracheal intubation using the conventional MC and the C‐MAC VL produces comparable overall hemodynamic responses during the early postintubation period. Repeated‐measures analysis demonstrated significant temporal changes in hemodynamic parameters following airway instrumentation but no significant group or group–time interaction effects between the two devices. The C‐MAC VL provided significantly better glottic visualization and a numerically higher first‐attempt intubation success rate, which may be advantageous when a difficult airway is anticipated. These findings suggest that both devices can be effectively used for airway management in this clinical setting, and device selection should be guided by operator experience, airway characteristics, and clinical context.

## Author Contributions

Sehrish Khan: conceptualization, data curation, methodology, project administration, and writing–original draft.

Malika Hameed: conceptualization, formal analysis, methodology, supervision, visualization, writing–original draft, and writing–review and editing.

Waleed Razi Khan: conceptualization, data curation, methodology, and project administration.

Muhammad Saad Yousuf: conceptualization, data curation, methodology, supervision, and writing–review and editing.

Khalid Samad: conceptualization, data curation, methodology, project administration, supervision, visualization, and writing–review and editing.

## Funding

No external funding sources were used.

## Disclosure

Presentation of the abstract in 4th National Anaesthesia Research Symposium: Enhancing the Quality of Anaesthesia Research in the Developing World, September 8‐10, 2023, Pakistan.

## Ethics Statement

The study was approved by ethics review committee of The Aga Khan University (approval no: 2020‐3278‐9046).

## Conflicts of Interest

The authors declare no conflicts of interest.

## Data Availability

The data that support the findings of this study are available from the corresponding author upon reasonable request.
